# The efficacy of ketamine in total knee arthroplasty: a randomized controlled trial protocol

**DOI:** 10.1097/MD.0000000000020645

**Published:** 2020-06-12

**Authors:** Jing Chen, Wei Hu, Shu-Ming Li, Xiao-Lin Li, Zhan-Min Yang

**Affiliations:** a Department of Orthopedics, Aerospace Center Hospital, Beijing; b Department of Orthopedics, Ningxia Armed Police Corps Hospital, Ningxia Hui Autonomous Region; c Department of Anesthesiology, Aerospace Center Hospital, Beijing, China.

**Keywords:** ketamine, pain control, randomized controlled trial, study protocol, total knee arthroplasty

## Abstract

**Background:**

Appropriate pain management is essential to improve the postoperative recovery after total knee arthroplasty (TKA). There is a paucity of literature on ketamine for TKA procedures. The aim of this study was to evaluate the analgesic efficacy of ketamine in patients undergoing primary TKA.

**Methods:**

This study was designed as a prospective, double blind, single center, randomized controlled trial. The participants were randomly assigned to either the ketamine or placebo groups, using a set of random numbers for the allocation sequence. All patients underwent TKA without patella resurfacing under spinal anesthesia. Preoperative workup, surgical technique, and postoperative management were standardized for all the patients. The primary outcome of this noninferiority study is opioid consumption within the first 24 hours following surgery, pain scores, distance ambulated, patient satisfaction, length of hospital stay, and complications.

**Results:**

The results of this study were expected to provide useful information on the effectiveness and safety of ketamine for immediate postoperative analgesia after TKA surgery.

**Trial registration:**

This study protocol was registered in Research Registry (researchregistry5575).

## 1 Introduction

Total knee arthroplasty (TKA) is a common surgical procedure that can cause severe postoperative pain. Postoperative pain management in patients undergoing TKA is a challenging issue that can optimize the treatment plan, reduce side effects, save health care resources, and increase patient satisfaction.^[1–3]^ Severe postoperative pain can impede initiation of an early rehabilitation program following TKA, which, in turn, can delay the range of motion at the knee joint and increase the risk of thrombotic complications, such as deep venous thrombosis and pulmonary embolism.^[4]^ Additionally, prolonged duration of hospitalization may increase more medical expenses. Therefore, pain management after TKA is an important issue for surgeons and patients to recognize.

Opioids are effective for acute postoperative pain after TKA. Persistent opioid use frequently follows surgery, so reducing postsurgical pain via opioid-sparing techniques can have long-term benefit. Preventive analgesia aims to reduce postoperative pain and analgesic consumption by combining multimodal analgesic therapies. Ketamine inhibits N-methyl-d-aspartate (NMDA) receptor activation and attenuates wind-up and central sensitization associated with hyperalgesia, opioid tolerance, and chronic pain.^[5–7]^

Ketamine is a phencyclidine derivative, and acts as a noncompetitive NMDA receptor antagonist.^[7]^ Ketamine, given at sub-anesthetic doses, has been shown to modulate nociceptive hypersensitization through its antagonist effects on NMDA receptors by blocking pain signaling input.^[8]^ There is significant evidence supporting the use of ketamine as an adjunct, to elicit the reduction in hypersensitization specifically when combined with low-dose morphine.^[9–12]^ Numerous publications state that adjuvant ketamine reduces pain and opioid consumption postoperatively.^[13,14]^ Although there is a paucity of literature on ketamine for orthopedic procedures, there are a few studies on intraoperative subanesthetic ketamine that are either of small sample sizes or performed with general anesthesia. As general anesthesia has been largely replaced by neuraxial anesthesia, it is important to evaluate ketamine in this setting.

The present study is a prospective, randomized, double blinded trial determining the efficacy of sub-anesthetic dosing of ketamine during TKA on postoperative pain and narcotic consumption in patients receiving spinal anesthesia. We hypothesized that the addition of intravenous ketamine to standard opioid analgesia would reduce pain scores and overall morphine equivalent consumption.

## 2 Material and method

This study was approved by the Institutional Review Board in our hospital and written informed consent was obtained from all subjects participating in the trial. The trial was also registered at the Research Registry (researchregistry5575). It was carried out in accordance with the principles of the Helsinki Declaration. Data are presented according to the CONSORT statement.

### 2.1 Study participants

Inclusion criteria were patients between 18 and 85 years old undergoing unilateral, primary TKA for osteoarthritis. Patients with the following conditions were excluded: body mass index greater than 40 kg/m^2^ due to patient selection, contraindication or allergy to opioid pain medication, any preoperative ketamine, and chronic narcotic usage (>10 mg systemic morphine equivalents daily). Furthermore, patients with an ejection fraction less than 30%, creatinine clearance less than 30 mL/min, chronic liver disease, any neurologic or psychiatric disorder (including bipolar post-traumatic stress disorder, schizophrenia), and alcohol abuse were excluded as these are contraindications for the use of ketamine. Additionally, patients with prior surgery on the ipsilateral knee within 6 months, simultaneous bilateral arthroplasty, and those who received general anesthesia or a nerve block were excluded.

### 2.2 Randomization and blinding

**Figure 1:**
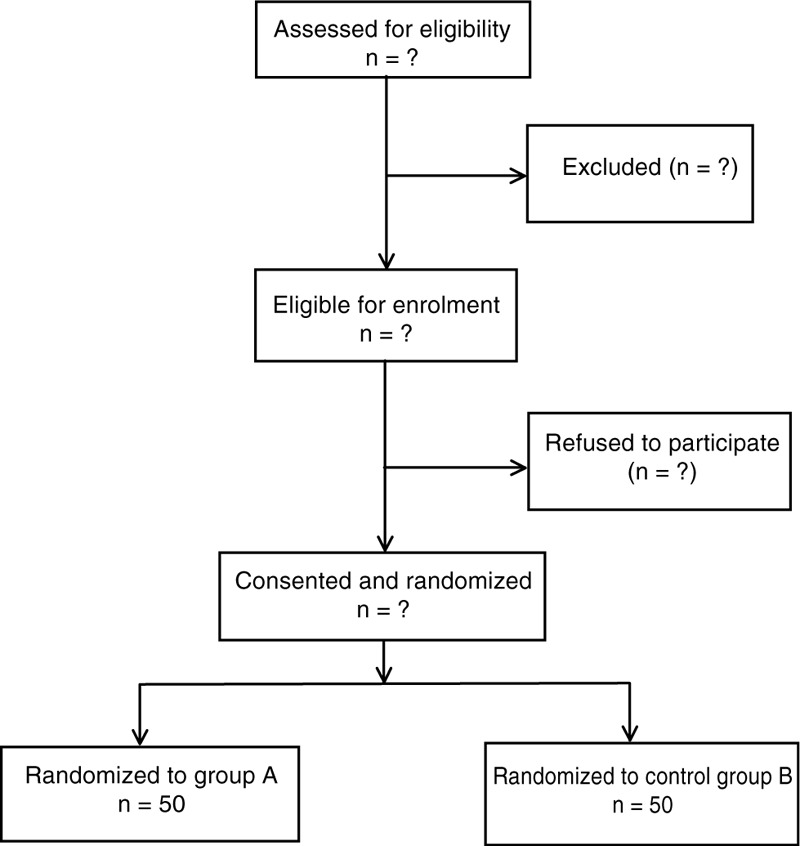
Flow diagram of the study.

An equal number of envelopes for each treatment group were prepared using a computerized random number generator by a study assistant who did not take part in any subsequent part of the study, and was not in contact with the rest of the study team throughout the entire study duration. He prepared 100 identical sequentially numbered, opaque, sealed, and stapled envelopes; 50 envelopes contained instructions for mixing solutions for Group A, and the other 50 for Group B. The envelopes were kept in a file with the principal investigator (Fig. 1).

After a patient was recruited by a member of the study team, a second study assistant selected an envelope randomly from the file and mixed the study solution according to the instructions in the envelope as per the patient's weight. He then resealed the envelope, placed it in a different file, and gave the study solutions labeled with the patient identity code (the same as that on the selected envelope) to the anesthesiologist in charge; thereafter, he did not partake in any subsequent part of the study. Both ketamine and normal saline study solutions had the same physical properties: clear liquids with no distinctive odor. Therefore, they could not be identified or differentiated by sight or smell by anyone in the operating theatre. The allocation sequence was concealed from both the study team and study assistants. The blinded study team labeled the data collection sheet with the patient identity code, and, in the operating theatre, verified the proper timing of study solution administration.

### 2.3 Surgery detail

All TKAs in this study were carried out by either the senior author, or by fellows under his direct supervision. A tourniquet was used in all cases, spine anesthesia was administered to each patient before incision, and the operative knee was prepared and draped in a conventional sterile fashion. All components were cemented with a conventional technique using antibiotic cement without any patient-specific instrumentation or robotic aide. The implants utilized were beaded periapatite-coated femoral, tibial, and patellar components (Triathlon Total Knee System; Stryker Orthopaedics, Mahwah, NJ).

### 2.4 Intervention

In patients assigned to the Group A, 0.05 mL/kg of the blinded test solution (ie, ketamine 0.5 mg/kg) was given Intravenous (IV) over 2 minutes just after the orotracheal intubation and before the skin incision. The initial bolus was followed by a maintenance IV infusion of 3 ug·kg^−1^·min^−1^ of ketamine that was continued until the patient emerged from anesthesia. Subsequently, the infusion rate was reduced to 1.5 ug·kg^−1^·min^−1^ and maintained for 48 hours. Patients allocated to the Group B were given identical volumes of saline.

### 2.5 Outcome measures

The primary outcome of this noninferiority study is opioid consumption within the first 24 hours following surgery. Subjects receive standardized postoperative multimodal analgesics. Supplemental oxycodone 5 to 15 mg every 3 hours for pain is available to each subject. Nurses administer a 5-mg oxycodone tablet for numerical rating scale (NRS) scores of 1 to 3, 10 mg for NRS scores of 4 to 6, and 15 mg for NRS scores of 7 to 10. Oral hydromorphone is substituted for oxycodone in the instances of patient allergy or intolerance. Rescue analgesia is available with IV hydromorphone 0.5 mg every 1 hour as needed for pain of greater than NRS of 7 when refractory to oral opioids. Opioid consumption during the first 48 hours postoperatively is retrieved from the electronic medical record and convert to IV morphine equivalents for analysis. The average and worst NRS pain scores at rest and with activity are determined by blinded investigators at 24 and 48 hours postoperatively.

Secondary outcomes included pain scores, distance ambulated, patient satisfaction, length of hospital stay, and complications. Length of hospital stay was calculated by measuring the time from the completion of surgery through discharge for each patient.

### 2.6 Statistical analysis

The statistical analysis was performed with the Statistical Package for Social Sciences (SPSS for Windows, release 12.0; SPSS Inc., Chicago, IL). Normal distribution was verified using the Kolmogorov–Smirnov test. Continuous variables were presented as median and interquartile range [Q25, Q75], or mean ± standard deviation according to distribution. Categorical parameters were expressed as number (%). The normal distributed numerical variables (NRS scores, distance ambulated, patient satisfaction, and length of hospital stay) were analyzed by Student *t* test. If the numerical variable has a nonnormal distribution or unequal variance, the Wilcoxon Mann–Whitney *U* test was used (American Society of Anesthesiologists grade); Pearson Chi-squared test or Fisher exact test was used to analyze the qualitative variable (complications). The nature of the hypothesis testing was 2-tailed, and a *P*-value < .05 was considered statistically significant.

### 2.7 Sample size calculation

The sample size was determined for the primary endpoint. According to the results of our previous study, the postoperative NRS score for nausea was 2.16 in the control group. We anticipated a difference of 0.72 in the NRS score. With a power of 0.90 and significance level of 0.05, the required sample size was calculated as 50 in each arm. Considering possible exclusion, we decided to include 60 patients in each group.

## 3 Discussion

TKA generates substantial postoperative pain. Although various effective interventions including femoral nerve block, peri-articular injection, adductor canal block, nonsteroid anti-inflammatory drug, and epidural morphine have been used for pain control after TKA, the optimal analgesia still remains a subject of debate. Recently, the use of ketamine has been popularized in the surgical field and exhibits improved outcomes for analgesia. Singh et al reported that preemptive ketamine has a definitive role in reducing postoperative pain and analgesic requirement in patients undergoing laparoscopic cholecystectomy.^[15]^ Becke et al demonstrated that intraoperative low dose ketamine had no effect on pain relief and morphine consumption during the first 72 hour after surgery.^[16]^ However, recent published articles reflected mixed results in TKA patients. Therefore, we performed a randomized controlled trial to evaluate the efficacy and safety of ketamine for reducing pain after TKA.

The present research seeks to compare the effect of ketamine on the postoperative analgesic effect and early rehabilitation in patients undergoing TKA. Three potential limitations to this study were identified. First, NRS pain was recorded by nursing pain assessment records in hospital and thus pain measurements are based on the frequency and accuracy of documentation in the medical record. The second limitation was the small sample size used in this study. Replication of this study on a multi-institutional level would provide less variation between experimental groups and simultaneously increase the reliability and generalizability of the results. Finally, having the same surgeon for all procedures in this study was both an advantage and disadvantage. It is advantageous for consistency and internal validity; however, it may limit the reproducibility of this study.

## Author contributions

**Conceptualization:** Jing Chen, Wei Hu.

**Data curation:** Shu-Ming Li.

**Formal analysis:** Jing Chen, Wei Hu.

**Funding acquisition:** Xiao-Lin Li.

**Investigation:** Jing Chen, Wei Hu.

**Methodology:** Zhan-Min Yang.

**Resources:** Zhan-Min Yang.

**Software:** Xiao-Lin Li.

**Supervision:** Zhan-Min Yang.

**Validation:** Shu-Ming Li.

**Visualization:** Shu-Ming Li.

**Writing – original draft:** Jing Chen, Wei Hu

**Writing – review & editing:** Zhan-Min Yang.
